# Honeybee cluster—not insulation but stressful heat sink

**DOI:** 10.1098/rsif.2023.0488

**Published:** 2023-11-22

**Authors:** Derek Mitchell

**Affiliations:** ^1^ Institute of Thermofluids, School of Mechanical Engineering University of Leeds, Leeds, UK; ^2^ Eigentek, Tadley, Hampshire RG26 3ED, UK

**Keywords:** *Apis mellifera*, convection, welfare, climate, ethics, Extended phenotype

## Abstract

Since the early twentieth century, the outer layer (mantle) of honeybees (*Apis mellifera*) in the winter cluster has been said to insulate the cluster core. This has encouraged enforced clustering, by the beekeepers' dominant use of inadequately insulated hives and, in North America, refrigeration. This is often seen as a benign or even a necessary process, with beekeeping and academic research considering these conditions of extreme heat loss, compared with the honeybee's natural habitat, as natural and normal. By using porous material correlations, analysis of previous findings and a model of a cluster within a hive in a landscape that implements convection, conduction and radiation, we show that a honeybee colony increases in thermal conductivity, on transition from pre-cluster to dense mantle, by a factor of approximately 2, and insulation *R*-value can decrease by more than 11. These results show that the mantle does not act like insulation and that clustering is not benign, but instead is an evolutionary behavioural reaction to an existential threat that results in increased cold and exertion stress. Thus the attitude to forced clustering, i.e. deliberately provoking a stressful survival behaviour, needs revision as avoidable forced stress upon animals may be regarded as cruel.

## 1. Introduction

**Figure 1.**
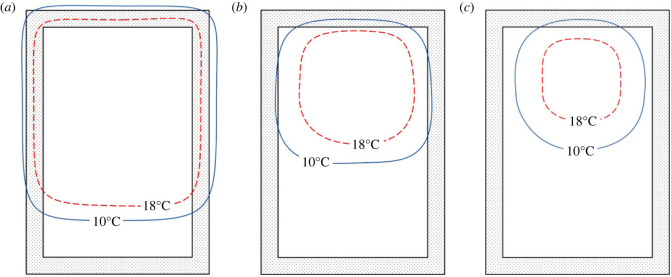
Clustering process from Stabentheiner *et al.* [4] and Owens [5] (*a*) pre-cluster, (*b*) low-density cluster and (*c*) dense cluster.

Honeybee colonies (*Apis mellifera*) overwinter in cavities keeping at least some of their number above 18°C [1]⁠ throughout the year in a wide range of climates that include −40°C winters. Human experience of their overwintering behaviour is almost exclusively by observation in thin walled (19 mm) wooden hives of very different thermal properties [2] to their preferred natural habitat of tree hollows e.g. thermal conductance of these hives can be up to seven times higher than tree hollows [3]⁠. In these hives, on warm days the honeybees are observed distributed about the hive engaged in various activities. On very cold days they form a cluster (figure 1), a series of dense discs of honeybees between the combs, the outline of the discs conforming to a rough spheroid (figure 5). The centres of these discs (core) are less dense and at a higher temperature (20°C to 34°C), producing almost all of the cluster heat [4]⁠. The outer layers of the cluster (mantle) that fall below 18°C generate little metabolic heat. Those honeybees on the periphery of the cluster that fall below 10°C must move inwards or will eventually die and fall from the cluster. This gives the surface temperature of the cluster a lower limit of approximately 10°C.

Research into the heat transfer of the honeybees within their nests has been limited to applying insulation to hives. These have been conducted:

1. without quantitatively measuring its heat transfer impact (apart from sometimes quoting the *R*-value of the sheet stock used [6], which is insufficient on its own see §2);
2. with the absence of realistic consideration of the heat transfer impact of apertures [7], including (i) not mentioning their presence or dimensions, (ii) quoting inadequate experiments [8];
3. by omitting quantitative comparison with the honeybees evolutionary preferred abode; and
4. failing to determine if the treatment has heat transfer significance compared with the control.

There are a few exceptions, e.g. Villumstad [9] measuring the thermal conductance of the hives.

The level of application of heat transfer experimental rigour and knowledge has led to an inability to make valid comparisons of the hives employed, and consequently, ambiguous or contradictory results [6,10]. When these results are combined with mixed support from academics [11,12] and the classification of the mantle as insulation [4,13], it has enabled poor uptake of permanently or intermittently insulated hives. This is despite many acknowledging the stress it causes. In addition, it has enabled the academic practice of employing high heat-loss hives as surrogates for honeybee preferred abodes. As research into honeybees and their behaviour inside cavities that have heat transfer similar to their natural abode is extremely rare [2], the validity of using hives as surrogates is uncertain.

From 1914, the mantle has been described as insulation [11,13–20]. This ‘insulation’ has been part of the rationale behind using high-conductance hives, in both peer-reviewed academic research and bee keeping [11,20] and the increasing North American practice (currently Southern California US to Canada [21]) of refrigeration. This applies an ambient temperature of approximately 4°C to colonies for winter and to force brood breaks [22,23], originally for economic reasons, and recently for varroa control. The survival of the colony in these circumstances is reliant on having large honeybee numbers and considerable metabolic heating, consuming up to 60 kg of honey in the most adverse conditions [24]. The low temperature differential between the internal space near the hive surface and the outside environment being attributed to the ‘honeybee heats the cluster not the hive’ rather than the high conductance of the hive compared with the rest of the system. This has led to high heat loss being seen as benign and/or beneficial [11,20,24] and use of alternatives as an illogical or emotional response [20].

In the apicultural literature, some questions remain open:

— **What constitutes insulation?** Any material causing a temperature difference, reductions in surface area and reductions in metabolism have all been termed ‘insulation’ [11,16,25].⁠ This and other qualitative usages differ from the more precise quantitative definition of insulation i.e. thermal insulance factor or *R*-value; the ratio of temperature difference to rate of heat transfer per unit area (heat flux) [26].
— **Convection or conduction?** The conduction or convection of pre-cluster state (i.e. not closely gathered into discs, figure 1), mantle and core have been, with one exception [27]⁠, assumed rather than analysed, measured or modelled. The most frequent assumption being: in all situations convection is dominant.
— **Where is the metabolic heat coming from?** The heat has been variously assumed to be coming from the mantle [28]⁠ and the core, with later infrared studies [4,29]⁠ placing source as the regions of the cluster above 18°C, i.e. the core, where individual bees, metabolizing sugars from honey, undergo a limited period of vigorous exertion of their thorax flight muscles before eventually becoming ectothermic and returning to the mantle and temperatures close to 10°C.
— **If and how does the hive body conductance, the air in the cavity and surrounding landscape contribute?** This has been largely ignored with the exception of one experimental [3]⁠ and one computational fluid dynamics (CFD) study [27]⁠, neither of which used a radiative landscape model.
— **If and how do** **honeybee** **bodies and hair contribute?** The quantitative thermal properties of the honeybee bodies and hair have either been ignored, used unrealistically [28]⁠ and/or *ad hoc* honeybee density to thermal conductivity [30,31]⁠ relations used rather than published engineering models.

## 2. Approach

We will proceed by:

— establishing criteria for the mantle being insulation or heat sink;
— determining the roles of conduction or convection or radiation in the core, mantle and pre-cluster heat transfer; and
— evaluating whether the above criteria are met, including the contributions from the hive, landscape, honeybee bodies and hair.

### 2.1. Insulation criteria

First, in colloquial speech, ‘insulation’ occurs when insulation substance is added, it results in reduced heat loss rate, and the opposite with ‘heat sink’. In other words: attach a bigger heat sink it will increase heat flow, if wrapped in thicker insulation material the object will have reduced heat flow. This qualitative criteria for being an insulator or heat sink can be expressed quantitatively as the sign of the slope $\left(\delta \dot{q}/\delta r\right)$ of the graph of heat transfer rate $\left(\dot{q}\right)$ versus cluster size (*r*), e.g. equation (2.1).

2.1 $$\begin{array}{r} \frac{\delta \dot{q}}{\delta r}\;>\;0\Rightarrow \mathrm{insulator}\;\frac{\delta \dot{q}}{\delta r}\;<\;0\Rightarrow \mathrm{heatsink}\;\;\;\mathrm{Criteria}\;1. \end{array}$$

Second, insulation can refer to a ‘more insulating material', i.e. a decrease in thermal conductivity of the substance, e.g. interlocking hairs on honeybee bodies have been described as ‘increasing the insulation’ as the bees get closer. For clustering to be termed insulation in this definition the effective conductivity *k*_eff_ of the bee/air mixture should decrease as the clustering progresses. The degree of clustering *Γ* is related to the porosity of the mantle *φ*, i.e. zero clustering (*Γ* = 0) when the mantle has the pre-cluster porosity *φ*_P_ and maximum clustering (*Γ* = 1) when the bees are tightly packed together (porosity= *φ*_0_), i.e. maximum clustering should accompany minimum mantle conductivity *k*_mantle_ if the mantel is insulation (equation (2.3)) and minimum clustering if a heat sink as per equation (2.3).

2.2 $$\Gamma =\frac{\varphi_{P}-\varphi }{\varphi_{P}-\varphi_{0}}$$

and

2.3 $$\begin{array}{rl} \frac{\delta k_{\mathrm{mantle}}}{\delta \Gamma }<0 & \Rightarrow \mathrm{insulator}\;\\ \frac{\delta k_{\mathrm{mantle}}}{\delta \Gamma }>0 & \Rightarrow \mathrm{heatsink}\;\;\mathrm{Criteria}\;2. \end{array}$$

Third, insulation can mean the application of material with lower thermal conductivity than that of the item being insulated. In this case we need to compare the thermal conductivity of the core and the mantle, i.e. equation (2.4).

2.4 $$\begin{array}{rl} k_{\mathrm{mantle}}<k_{\mathrm{core}} & \Rightarrow \mathrm{insulator}\;\\ k_{\mathrm{mantle}}>k_{\mathrm{core}} & \Rightarrow \mathrm{heatsink}\;\;\mathrm{Criteria}\;3. \end{array}$$

Fourth, using the definition of thermal insulance (*R*-value), temperature difference per unit of heat flux in equation (2.5), we can then test if *R*-value increases with clustering, i.e. the gradient of *R*-value with respect to clustering.

2.5 $$R_{\mathrm{value}}=\frac{T_{\mathrm{Core}}-T_{\mathrm{Mantle}}}{\dot{q}/A}$$

and

2.6 $$\frac{\delta R_{\mathrm{value}}}{\delta \Gamma }>0\Rightarrow \mathrm{insulator}\;\;\;\mathrm{Criteria}\;4.$$

### 2.2. Conduction or convection or radiation

Common building insulation (styrofoam, rock wool) and cold climate clothing rely on gases to perform the actual insulation, but they need to keep the gases still or nearly still to prevent a high rate of heat transfer via convection. When this is achieved we get thermal conductivities (0.025 W m^−1^ K^−1^) an order of magnitude lower (better) than the best non-metallic solid (e.g. plastics 0.12–0.5 W m^−1^ K^−1^, wool fibre 0.5 W m^−1^ K^−1^) [32]⁠ and the body of a honeybee (0.5 W m^−1^ K^−1^; see Methods). The thermal resistance of a collection of objects with gaps between them is dependent on the distance between the objects, their effective diameter and the thickness of the object collection [33]⁠.

**Figure 2.**
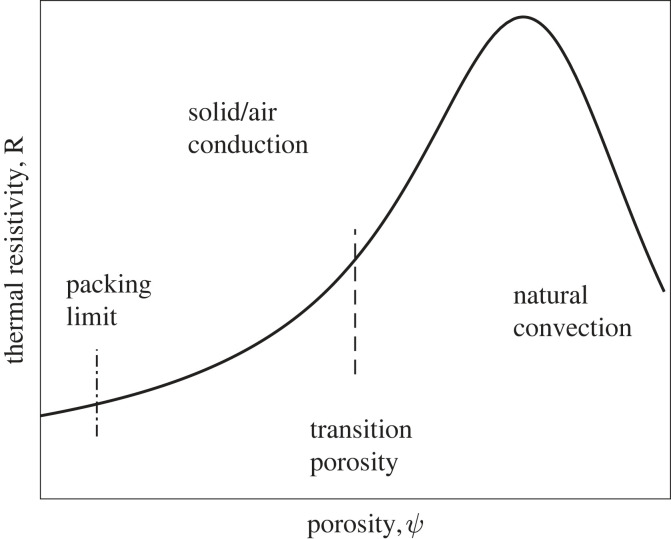
Typical thermal resistance versus porosity.

When the distance between the objects is relatively large (porosity close to 1) then convection currents, set up within the air between the objects, dominate the heat transfer. These currents decrease as the porosity decreases. When the porosity falls below a particular value (dependent on the gas properties, temperature differences, geometry, etc.), the convection currents stop, then heat transfer takes place by conduction only, both though the air and importantly, the objects. As the porosity decreases further, the thermal conductivity becomes more like the object and less like that of air. If the objects have a high conductivity compared with air, it results in a variation of thermal resistance to porosity like that shown in figure 2, where the thermal resistance peaks near the cessation of convection currents and falls on either side, at lower porosities due to conduction, at higher porosities due to convection. The porosity has a lower limit determined by geometry of the objects. For a mixture of cylinders of two different sizes, e.g. *k*_mantle_ honeybees and hair, this is between 0.1 and 0.01 depending on the mixture.

This transition porosity can be determined by experiment, CFD or by analysis. The latter is where a dimensionless number (Rayleigh number) is evaluated for a porous system or part system and if above a threshold indicates natural convection will start. One CFD analysis has shown that the average initial winter population of *Apis mellifera mellifera* [34] in a British National hive complete with combs may be sufficient to suppress natural convection⁠ [27]⁠. One can visualize this as when the honeybees are evenly distributed throughout the nest, the resistance of the circulation path of convecting air is equivalent to a 0.8 m long, 20 mm wide tube, half filled with small objects, with circulation propelled only by the tiny amount of buoyancy created by a few degrees of temperature difference.

If we can treat the mixtures of honeybees and air, both pre-cluster and cluster as solids, then we can estimate the effective conductivity (*k*_eff_) of any bee/air mixture given the conductivity of bee bodies (*k*_body_) and air (*k*_air_) using the effective medium theory (EMT) correlation described by Carson *et al.* [33]⁠ and not the unrealistic models employed by others [28,35].

The literature on porous solid heat transfer [33]⁠ shows that determining the effective conductivity of the combination of the gas and solid is complex even when convection is eliminated. It has been shown that variation of conductivity with porosity is likely to be the EMT (equation (2.7)).

2.7 $$\begin{array}{rl} k_{\mathrm{eff}} & =\frac{1}{4}((3\varphi -1)k_{\mathrm{air}}+[3(1-\varphi )-1]k_{B})\;\\ & \;+\sqrt{{\left\{(3\varphi -1)k_{\mathrm{air}}+[3(1-\varphi )-1]k_{B}\right\}}^{2}+8k_{\mathrm{air}}k_{B}})\; \end{array}.$$

For the known conductivities of air and honeybee bodies, this gives the relationship between effective conductivity and porosity.

**Figure 3.**
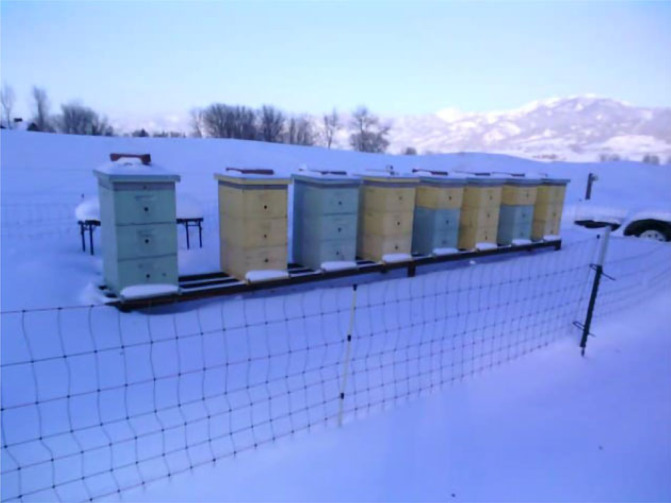
Hives in a bare cold landscape (Scott Hall).

**Figure 4.**
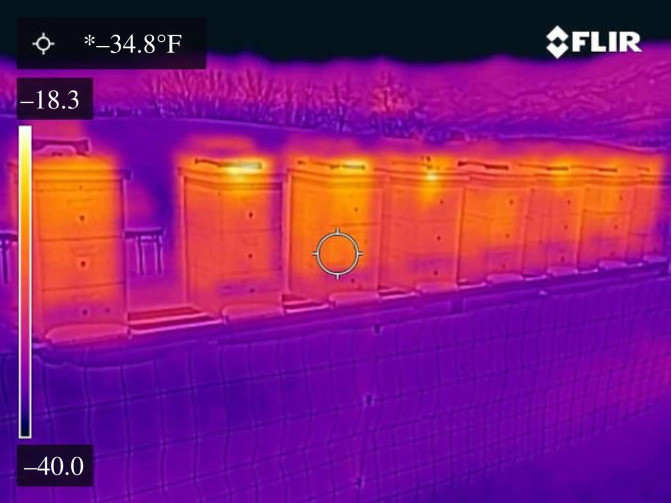
Hives in a bare landscape via infrared (FLIR C5, Scott Hall).

The thermal system of a honeybee cluster within a hive in a realistic landscape is a complex one in which factors such as radiation and effective sky temperature, not often considered, can be significant; however, we can readily produce a reasonably detailed approximation. This is where we consider the cluster to be a solid sphere inside a box set above ground in a cold bare landscape with a clear sky at low humidity, e.g. figures 3 and 4. In this scenario, it is sufficiently cold for the sphere of honeybees to have a surface temperature of 10°C regardless of size, as observed by various researchers [4]⁠.

## 3. Methods

### 3.1. Conduction or convection transition

The critical Rayleigh number in a natural convection system determines whether natural convection can occur. If the Rayleigh number is above the critical number, convection starts in addition to conduction, below this number only conduction occurs. This involves calculation of both permeability and the diffusivity of the bee/air mixtures. When the honeybees are far apart, the total permeability *K*_T_ is made up of the permeability around the space enveloped by bee bodies and hair *K*_BH_ (symbols are given in table 1). As honeybees come together it changes into permeability through hair with bee bodies in it *K*_B_. The changeover is governed by porosity around bee and hair envelopes φ_BH_ as shown in equation (3.1).

3.1 $$K_{T}=K_{\mathrm{BH}}+(1-\varphi_{\mathrm{BH}})K_{B}.$$

**Table 1.** Symbols used in equations.

symbol	units	description
$c_{1}$ to $c_{4}$	—	constants for sphere–cube convection correlation [36]⁠
$d_{B},\;d_{BH},\;d_{h}$	m	diameters of individual honeybee body, body and hair envelope and hair
${\bar{d}}_{B},\;{\bar{d}}_{BH}$	m	Sauter mean diameters of individual bee body, body and hair envelope
$\;f_{\mathrm{ground}}$	—	view factor radiated hive outer to ground
$\;f_{\mathrm{sky}}$	—	view factor hive to sky
${\bar{h}}_{a(\mathrm{conv})}$	W m^−2^ K^−1^	average heat transfer coefficient of total hive outer surface
${\bar{h}}_{\mathrm{bottom}}$	W m^−2^ K^−1^	average heat transfer coefficient of hive outer bottom surface
${\bar{h}}_{Li}$	W m^−2^ K^−1^	average heat transfer coefficient of gap between mantle and hive interior
${\bar{h}}_{\mathrm{side}}$	W m^−2^ K^−1^	average heat transfer coefficient of hive outer side surface
${\bar{h}}_{\mathrm{top}}$	W m^−2^ K^−1^	average heat transfer coefficient of hive outer top surface
*g*	m s^−2^	acceleration of gravity 9.81 m s^−2^
$k_{\mathrm{air}}$	W m^−1^ K^−1^	thermal conductivity of air
$k_{\mathrm{hive}}$	W m^−1^ K^−1^	thermal conductivity for hive
$k_{\mathrm{eff}}$	W m^−1^ K^−1^	effective thermal conductivity of honeybees in cluster mantle
$k_{B}$	W m^−1^ K^−1^	effective thermal conductivity of a honeybee body
$l_{B},\;l_{BH},\;l_{h}$	m	length of individual honeybee body, body and hair envelope and hair
$m_{B}$	kg	mass of individual honeybee
$\;p_{h}$	—	plumosity of honeybee hair
${\dot{q}}_{g(\mathrm{rad})}$	W	heat transfer rate radiated from hive outer to ground
${\dot{q}}_{\mathrm{inner}(\mathrm{conv})}$	W	heat transfer rate hive inner convection
${\dot{q}}_{\mathrm{hive}(\mathrm{conduct})}$	W	heat transfer rate between hive inner and outer surfaces by conduction
${\dot{q}}_{\mathrm{mantle}}$	W	total heat transfer rate from mantle
${\dot{q}}_{\mathrm{thermal}}$	W	sky downward heat flux
${\dot{q}}_{\mathrm{sky}(\mathrm{rad})}$	W	heat flux radiated from hive outer to sky
${\dot{q}}_{\mathrm{inner}(\mathrm{rad})}$	W	heat transfer rate outer radiation
${\dot{q}}_{\mathrm{air}(\mathrm{conv})}$	W	heat transfer rate outer convection
$r_{\mathrm{eff}}$	m	effective radius of hive cavity
$r_{\mathrm{mantle}}$	m	radius of mantle
$x_{a},\;y_{a},\;z_{a}$	m	dimension of hive *a* = inner or outer
$A_{B},\;A_{BH},\;A_{h}$	m^2^	surface areas of individual honeybee body, body and hair envelope and hair
$A_{\mathrm{side}}$	m^2^	total area of hive outer vertical sides
*A*_bottom_	m^2^	area of hive outer bottom surface
*A*_top_	m^2^	area of hive outer top surface
*A*_outer_	m^2^	total area of hive outer surfaces
*A*_inner_	m^2^	total area of hive inner surfaces
*A*_mantle_	m^2^	area of mantle outer surface
*C*	—	cloud cover coefficient
$Cp_{\mathrm{air}},\;Cp_{B}$	J kg^−1^ K^−1^	heat capacities of air and honeybees
*H*	m	vertical dimension of the core
*L*_x_	m	characteristic length x
*L*_bottom_	m	characteristic length of hive outer bottom surface
*L*_i_	m	characteristic length of gap between mantle and hive interior
*L*_side_	m	characteristic length of hive outer vertical side surfaces
*L*_top_	m	characteristic length of hive outer top surface
$\bar{L}$	m	characterisitic length of honeybees between combs
*K*	—	cloud height coefficient
${\bar{Nu}}_{\mathrm{bottom}}$	—	average Nusselt number of hive outer bottom surface
${\bar{Nu}}_{\mathrm{Li}}$	—	average Nusselt number of hive mantle gap
${\bar{Nu}}_{\mathrm{side}}$	—	average Nusselt number of hive outer side surface
${\bar{Nu}}_{\mathrm{top}}$	—	average Nusselt number of hive outer top surface
Pr	—	Prandtl number of air
$Ra_{Li}$	—	Rayleigh number of hive mantle gap
$Ra_{Lx}$	—	Rayleigh number at characteristic length *L_x_*
$Ra_{L\mathrm{top}}$	—	Rayleigh number of hive top surface
$Ra_{L\mathrm{bottom}}$	—	Rayleigh number of hive bottom surface
$Ra_{L\mathrm{side}}$	—	Rayleigh number of hive side surfaces
$Ra_{\bar{L}}$	—	Rayleigh number of honeybees between combs
$Ra_{C}$	—	critical Rayleigh number typically approximately 40 for porous materials
$R_{\mathrm{value}}$	K m^2^ W^−1^	*R*-value thermal insulance [26]
$R_{H}$	—	relative humidity of air (0–1)
S	—	total conduction shape factor for hive [37]
$T_{\mathrm{air}}$	K	temperature of air
$T_{\mathrm{film}}$	K	temperature to calculate air properties
$T_{\mathrm{ground}}$	K	temperature of ground
$T_{\mathrm{hive}(\mathrm{inner})}$	K	temperature of hive inner surface
$T_{\mathrm{hive}(\mathrm{outer})}$	K	temperature of hive outer surface
$T_{\mathrm{mantle}}$	K	temperature of mantle outer surface
$T_{\mathrm{core}}$	K	temperature of core–mantle boundary
$T_{\mathrm{sky}}$	K	effective temperature of sky
$V_{B},\;V_{BH},\;V_{h}$	m^3^	volumes of individual honeybee body, body and hair envelope and hair
$\alpha_{T},\;\alpha_{B}$	m^2^ s^−1^	thermal diffusivity at temperature $T_{\mathrm{film}}$ and honeybee bodies
$\beta_{T}$	K^−1^	thermal expansion coefficient at temperature $T_{\mathrm{film}}$
$\varepsilon_{\mathrm{ground}}$	—	emissivity of ground typically 0.9
$\varepsilon_{\mathrm{inner}}$	—	emissivity of hive inner surface typically 0.9
$\varepsilon_{\mathrm{outer}}$	—	emissivity of hive outer surface typically 0.9
$\varepsilon_{\mathrm{mantle}}$	—	emissivity of mantle 0.9
$\varepsilon_{\mathrm{sky}}$	—	emissivity of sky 0.75
$\varphi_{B},\;\varphi_{BH}$	—	porosity of honeybees in mantle i.e. fraction of air
$\varphi_{0},\;\varphi_{P}$	—	porosity of honeybee when tightly packed in mantle, and pre-clustered
$\nu_{T}$	m^2^ s^−1^	kinematic viscosity at temperature $T_{\mathrm{film}}$
$\rho_{Ah}$	m^−2^	surface density of hairs on honeybee
$\rho_{B}$	kg m^−3^	density of honeybee
τ	m	hive wall thickness
$\chi_{Vh}$	—	volume ratio of hair on a honeybee
σ	kg s^−3^ K^−4^	Steffan–Boltzmann constant
Γ	—	degree of clustering

We can derive the terms in equation (3.1) by considering bee plus hair as a combined particle with length and diameter *l*_BH_, *d*_BH_ from the bee body and hair dimensions *l*_B_, *d*_B_, *l*_h_, *d*_h_.

We can then define the volumes and areas of the bee bodies, the combined particle of bee bodies plus hair *V*_BH_, *A*_BH_ and hairs *V*_h_, *A*_h_ as per equations (3.2–3.4).

3.2 $$l_{\mathrm{BH}}=l_{B}+2l_{h}\;d_{BH}=d_{B}+2l_{h},\;$$

3.3 $$V_{\mathrm{BH}}=\frac{\pi }{4}l_{\mathrm{BH}}d_{\mathrm{BH}}^{2}\;\;V_{B}=\frac{\pi }{4}l_{B}d_{B}^{2}\;\;V_{h}=\frac{\pi }{4}l_{h}d_{h}^{2},\;$$

3.4 $$\begin{array}{rl} & A_{\mathrm{BH}}=\pi l_{\mathrm{BH}}d_{\mathrm{BH}}+\frac{\pi }{2}d_{\mathrm{BH}}^{2}\;\;A_{B}=\pi l_{B}d_{B}+\frac{\pi }{2}d_{B}^{2}\;\;A_{h}=\pi l_{h}d_{h}+\frac{\pi }{2}d_{h}^{2}. \end{array}$$

φ_BH_ can then be derived from the porosity around bee bodies φ_B_ and *V*_BH_, *V*_B_ equation (3.5).

3.5 $$\varphi_{\mathrm{BH}}=1-\frac{(1-\varphi_{B})V_{\mathrm{BH}}}{V_{B}}\;\mathrm{where}\;\varphi_{\mathrm{BH}}\geq 0.$$

Using the volume fraction in equation (3.5), we can then derive the effective particles diameters using the Sauter mean and the cubic average as used by Glover [38]⁠ in equation (3.6) and equation (3.7).

3.6 $$\chi_{\mathrm{Vh}}=\frac{A_{B}p_{h}\rho_{\mathrm{Ah}}V_{h}}{V_{B}+A_{B}p_{h}\rho_{\mathrm{Ah}}V_{h}}$$

and

3.7 $${\bar{d}}_{\mathrm{BH}}=\frac{6V_{\mathrm{BH}}}{A_{\mathrm{BH}}}\;\;\;\;{\bar{d}}_{B}=6\left(\frac{V_{B}}{A_{B}}-\left(\frac{V_{B}}{A_{B}}-\frac{V_{h}}{A_{h}}\right)\chi_{\mathrm{Vh}}^{1/3}\right).$$

The different effective size for the permeabilities is shown in equation (3.8).

3.8 $$\begin{array}{rl} & K_{\mathrm{BH}}={\bar{d}}_{\mathrm{BH}}^{2}\frac{\varphi_{\mathrm{BH}}^{3}}{180{\left(1-\varphi_{\mathrm{BH}}\right)}^{2}}\;\;K_{B}={\bar{d}}_{B}^{2}\frac{\varphi_{B}^{3}}{180{\left(1-\varphi_{B}\right)}^{2}} \end{array}.$$

In equation (3.9) the diffusivity is derived according to Carson *et al.* [33]⁠ from the hairless size and porosity. The effective conductivity is calculated using the EMT from equation (2.7).

3.9 $$\alpha_{B}=\frac{k_{B}}{\rho_{B}Cp_{B}(1-\varphi_{B})+\rho_{\mathrm{air}}Cp_{\mathrm{air}}\varphi_{B}}\;\mathrm{where}\;\rho_{B}=\frac{m_{B}}{V_{B}}.$$

In equation (3.8) the permeability is derived according to Nield & Bejan [39] ⁠from the size and porosity including the honeybee hair.

The mantle can be treated as a porous volume heated allowing the Rayleigh number to be calculated using the permeability and diffusivity, the temperature difference and the characteristic length. Given the gap between the combs is significantly less than the size of the sum of the two boundary layers, the characteristic length may be interpreted as the gap. For this Rayleigh number, convection occurs when it exceeds a value of approximately 40.

3.10 $$Ra_{\bar{L}}=\frac{g\beta K_{T}(T_{0}-T_{1})\bar{L}}{\nu \alpha_{B}}.$$

### 3.2. Honeybee body, pre-cluster, core and mantle thermal conductivity

Ocko & Mahadevan [30] derived a conductivity (0.17 W m^−1^ K^−1^) at a known porosity (0.5) from an experiment by Southwick [40]⁠. Basak *et al.* [31]⁠ derived a conductivity (0.2 W m^−1^ K^−1^) from Heinrich [25]⁠ for a disordered packing of honeybees.

By using these experiments in bee/air mixture thermal conductivity [25,30,31,41]⁠ (table 3), equation (2.7), and approximating honeybees to sphero-cylinders of aspect ratio 3 (length 14 mm, diameter 5 mm), we can determine a bee body conductivity and use that to determine the conductivity of the bee/air mixtures from pre-clustered state to the densest possible mantle.

**Table 3.** Honeybee body thermal conductivity.

experimenter/s	experimental conductivity W m^−1^ K^−1^	experimental porosity	honeybee body conductivity W m^−1^ K^−1^
Basak, Heinrich, Abre	0.2	0.45	0.5012
Ocko, Southwick	0.17	0.5	0.5008

Using these pairs of values for conductivity and porosity and iteratively solving equation (2.7) [42]⁠, we can determine two values for honeybee body thermal conductivity. These values are within the range of experimental results for other types of solid flesh [43]⁠.

The porosity of the mantle when tightly clustered is made up of both bees and hair which can be approximated to cylinders and sphero-cylinders respectively. The limiting porosity therefore lies some where between 0.09 and 0.09^2^, i.e. 0.008 [38,44]⁠. The pre-clustered porosity is derived from CFD investigations into hive convection (0.5) [27].

From the above determined honeybee body conductivity, the conductivity of air, pre-cluster and mantle porosities, we can determine the thermal conductivity of the mantle and pre-cluster honeybees from equation (2.7).

### 3.3. Heat loss, mantle size ratio determination

**Figure 5.**
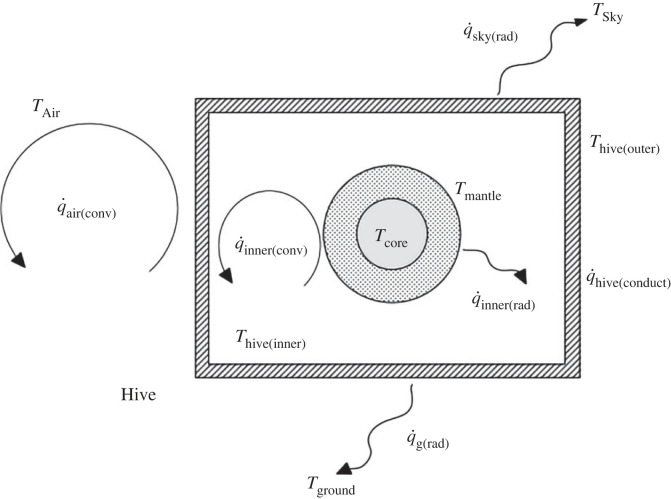
Cluster hive landscape schematic.

This considers the honeybee winter cluster as a sphere with its surface at a constant temperature inside, but not in contact with, a completely closed box above the ground, in a bare landscape with radiation convection and conduction (figure 5). The ground, air and sky are at potentially different temperatures. The energy of the cluster is transmitted to the hive interior surface via convection and radiation. It moves from the interior surface of the hive to the exterior surface by conduction. This is then convected into the air and radiated to the ground and sky. As the outside temperatures decrease the energy output of the colony increases and the surface area of the colony decreases as the colony contracts.

Conservation of energy implies the following: convection and radiation from hive outer surface to environment (equation (3.11)),

3.11 $${\dot{q}}_{\mathrm{mantle}}+({\dot{q}}_{\mathrm{air}(\mathrm{conv})}+{\dot{q}}_{g(\mathrm{rad})}+{\dot{q}}_{\mathrm{sky}(\mathrm{rad})})=0,$$

conduction through hive from hive inner surface to hive outer surface (equation 3.12),

3.12 $${\dot{q}}_{\mathrm{mantle}}+{\dot{q}}_{\mathrm{hive}(\mathrm{conduct})}=0,$$

convection and radiation from mantle surface to hive inner surface (equation 3.13),

3.13 $${\dot{q}}_{\mathrm{mantle}}+({\dot{q}}_{\mathrm{inner}(\mathrm{conv})}+{\dot{q}}_{\mathrm{inner}(\mathrm{rad})})=0.$$

This involves the iterative simultaneous solution of three equations (3.11, 3.12, 3.13) [42] for ${\dot{q}}_{\mathrm{mantle}},\;T_{\mathrm{hive}(\mathrm{outer})},\;T_{\mathrm{hive}(\mathrm{inner})}$ for values of *T*_air_, *r*_mantle_ when *T*_ground_, *T*_sky_ can be derived from *T*_air_.

The determination of the terms ${\dot{q}}_{\mathrm{air}(\mathrm{conv})},\;{\dot{q}}_{g(\mathrm{rad})},\;{\dot{q}}_{\mathrm{sky}(\mathrm{rad})},$ ${\dot{q}}_{\mathrm{hive}(\mathrm{conduct})},\;{\dot{q}}_{\mathrm{inner}(\mathrm{conv})},\;{\dot{q}}_{\mathrm{inner}(\mathrm{rad})}$ are described below.⁠

Hive outer surface to environment by convection heat flux ${\dot{q}}_{\mathrm{air}(\mathrm{conv})}$ is calculated for the sides, top and bottom separately assuming uniform surface temperature via calculation of the Rayleigh and Nusselt numbers in equations (3.14) to (3.26) using air properties *α*, *β*, *ν*, *Pr* at average temperature (equation 3.18) and the hive dimensions [45]⁠ (*x*_inner_ = 0.44, *y*_inner_ = 0.3, *z*_inner_ = 0.44, *τ* = 0.012)⁠

3.14 $$\begin{array}{r} x_{\mathrm{outer}}=x_{\mathrm{inner}}+\tau \end{array}\;\;\;y_{\mathrm{outer}}=y_{\mathrm{inner}}+\tau \;\;\;z_{\mathrm{outer}}=z_{\mathrm{inner}}+\tau ,\}$$

3.15 $$\begin{array}{r} A_{\mathrm{side}}=2z_{\mathrm{outer}}y_{\mathrm{outer}}+2x_{\mathrm{outer}}y_{\mathrm{outer}}\;\;A_{\mathrm{bottom}}=A_{\mathrm{top}}=y_{\mathrm{outer}}x_{\mathrm{outer}} \end{array},$$

3.16 $$A_{\mathrm{outer}}=A_{\mathrm{top}}+A_{\mathrm{bottom}}+A_{\mathrm{side}},\;$$

3.17 $$A_{\mathrm{inner}}=2x_{\mathrm{inner}}y_{\mathrm{inner}}\;+2x_{\mathrm{inner}}z_{\mathrm{inner}}+2y_{\mathrm{inner}}z_{\mathrm{inner}}\;,$$

3.18 $$T_{\mathrm{film}}=\frac{T_{\mathrm{hive}(\mathrm{outer})}+T_{\mathrm{air}}}{2},\;$$

3.19 $$Ra_{Lx}=\frac{g\beta_{T}(T_{\mathrm{hive}(\mathrm{outer})}-T_{\mathrm{air}})L_{x}^{3}}{\alpha_{T}\nu },\;$$

3.20 $$L_{\mathrm{top}}=L_{\mathrm{bottom}}=\frac{A_{\mathrm{top}}}{2x_{\mathrm{outer}}+2z_{\mathrm{outer}}},$$

3.21 $$\begin{array}{rl} & L_{\mathrm{side}}=z_{\mathrm{outer}}\;\;\;L_{\mathrm{top}}=L_{\mathrm{bottom}}=\frac{A_{\mathrm{top}}}{2x_{\mathrm{outer}}+2y_{\mathrm{outer}}} \end{array},$$

3.22 $${\bar{Nu}}_{\mathrm{top}}=0.27Ra_{L\mathrm{top}}^{1/4},$$

3.23 $${\bar{Nu}}_{\mathrm{bottom}}=0.15{Ra_{L\mathrm{bottom}}}^{1/3},$$

3.24 $${\bar{Nu}}_{\mathrm{side}}=0.68+\frac{0.670Ra_{L\mathrm{side}}^{1/4}}{{\left[1+{\left(0.492/Pr\right)}^{9/16}\right]}^{4/9}},\;$$

3.25 $$\begin{array}{rl} & {\bar{h}}_{\mathrm{top}}=\frac{{\bar{Nu}}_{\mathrm{top}}k_{\mathrm{air}}}{L_{\mathrm{top}}}\;\;{\bar{h}}_{\mathrm{bottom}}=\frac{{\bar{Nu}}_{\mathrm{bottom}}k_{\mathrm{air}}}{L_{\mathrm{bottom}}}\;\;\;{\bar{h}}_{\mathrm{side}}=\frac{{\bar{Nu}}_{\mathrm{side}}k_{\mathrm{air}}}{L_{\mathrm{side}}}\; \end{array},$$

3.26 $${\bar{h}}_{a(\mathrm{conv})}=\frac{4{\bar{h}}_{\mathrm{side}}A_{\mathrm{side}}+{\bar{h}}_{\mathrm{top}}A_{\mathrm{top}}+{\bar{h}}_{\mathrm{bottom}}A_{\mathrm{bottom}}}{4A_{\mathrm{side}}+A_{\mathrm{top}}+A_{\mathrm{bottom}}}$$

3.27 $$\;\;\begin{array}{c} {\dot{q}}_{\mathrm{air}(\mathrm{conv})}={\bar{h}}_{a(\mathrm{conv})}A_{\mathrm{outer}}(T_{\mathrm{hive}(\mathrm{outer})}-T_{\mathrm{air}})\; \end{array}.$$

Hive outer surface to environment by radiation to sky ${\dot{q}}_{\mathrm{sky}(\mathrm{rad})}$ and ground ${\dot{q}}_{g(\mathrm{rad})}$ heat fluxes are derived via equations (3.30, 3.31). Sky temperature is computed [46] for high (*K* = 0.06) cloudless (*C* = 0) and relative humidity of 1% (*R_H_* = 0.01) (equations 3.28, 3.29) with little or no shade (*f*_sky_ = *f*_ground_ = 0.5).⁠

3.28 $${\dot{q}}_{\mathrm{thermal}}=(1+KC^{2})8.78\times 10^{-13}T_{\mathrm{air}}^{5.852}{\left(100\times R_{H}\right)}^{0.07195},$$

3.29 $$T_{\mathrm{sky}}={\left(\frac{{\dot{q}}_{\mathrm{thermal}}}{\varepsilon_{sky}\sigma }\right)}^{1/4},$$

3.30 $${\dot{q}}_{\mathrm{sky}(\mathrm{rad})}=f_{\mathrm{sky}}\varepsilon_{\mathrm{outer}}\sigma A_{\mathrm{outer}}(T_{\mathrm{hive}(\mathrm{outer})}^{4}-T_{\mathrm{sky}}^{4})$$

3.31 $$\;\;{\dot{q}}_{g(\mathrm{rad})}=f_{\mathrm{ground}}\varepsilon_{\mathrm{outer}}\sigma A_{\mathrm{outer}}(T_{\mathrm{hive}(\mathrm{outer})}^{4}-T_{\mathrm{ground}}^{4}).$$

Hive inner surface to hive outer surface by conduction ${\dot{q}}_{\mathrm{hive}(\mathrm{conduct})}$ heat flux is derived using shape factors for a cuboid [37] in equation (3.32)⁠

3.32 $$S=\frac{A_{\mathrm{inner}}}{\tau }+2.16(x_{\mathrm{inner}}+y_{\mathrm{inner}}+z_{\mathrm{inner}})+1.22\tau$$

and

3.33 $${\dot{q}}_{\mathrm{hive}(\mathrm{conduct})}=S\;*\;k_{\mathrm{hive}}(T_{\mathrm{hive}(\mathrm{outer})}-T_{\mathrm{hive}(\mathrm{inner})}).$$

Mantle to hive interior surface by convection heat flux ${\dot{q}}_{inner(conv)}$ is computed using the correlations [36]⁠ in equations (3.34) to (3.41) using air properties *α*, *β*, *ν*, *Pr* at average temperature equation (3.37)

3.34 $$A_{\mathrm{mantle}}=4\pi r_{\mathrm{mantle}}^{2}\;,$$

3.35 $$r_{\mathrm{eff}}={\left(\frac{x_{\mathrm{inner}}y_{\mathrm{inner}}z_{\mathrm{inner}}}{4\pi /3}\right)}^{1/3},$$

3.36 $$L_{i}=r_{\mathrm{eff}}-r_{\mathrm{mantle}}\;,$$

3.37 $$T_{\mathrm{film}}=\frac{T_{\mathrm{hive}(\mathrm{inner})}+T_{\mathrm{mantle}}}{2},$$

3.38 $$Ra_{Li}=\frac{g\beta (T_{\mathrm{mantle}}-T_{\mathrm{hive}(\mathrm{inner})})L_{i}^{3}}{\alpha_{T}\nu_{T}},\;$$

3.39 $${\bar{Nu}}_{Li}=c_{1}Ra_{Li^{c_{2}}}{\left(\frac{L_{i}}{r_{\mathrm{mantle}}}\right)}^{c_{3}}Pr^{c_{4}},\;$$

3.40 $${\bar{h}}_{Li}=\frac{k_{air}{\bar{Nu}}_{Li}}{L_{i}}$$

3.41 $$\;\;{\dot{q}}_{\mathrm{inner}(\mathrm{conv})}={\bar{h}}_{Li}A_{\mathrm{mantle}}(T_{\mathrm{mantle}}-T_{\mathrm{hive}(\mathrm{inner})}).$$

Mantle to hive interior by radiation heat flux ${\dot{q}}_{\mathrm{inner}(\mathrm{rad})}$ is computed using the equation for concentric spheres [47]⁠ in equation (3.42).

3.42 $${\dot{q}}_{\mathrm{inner}(\mathrm{rad})}=\frac{\sigma A_{\mathrm{mantle}}(T_{\mathrm{mantle}}^{4}-T_{\mathrm{hive}(\mathrm{inner})}^{4})}{(1/\varepsilon_{\mathrm{mantle}})+(1-\varepsilon_{\mathrm{inner}}/\varepsilon_{\mathrm{inner}}){\left(r_{\mathrm{mantle}}/r_{\mathrm{eff}}\right)}^{2}}.$$

### 3.4. *R*-value analysis

In the published results of [5], the temperature contours inside a hive are shown for a colony clustering during falling external temperatures (figures 1 and 5). From the width of the 10°C contour one can estimate the external diameter of that colony mantle in that hive at varying external ambient temperatures. Using hive dimensions given in the publication and the model in §3.3 then one can calculate the metabolic heat production for a shaded hive. From the definition of thermal insulance or *R*-value in equation (2.5), and the known temperatures for the mantle external surface and core of brood-less clusters (10°C, 20°C), one can then determine the change in *R*-value. Also, from the model, we can determine how much of the core to ambient temperature difference is a result of the mantle, air gap between the mantle and the hive, the hive body and external surface heat transfer.

## 4. Results

### 4.1. Convection conduction transition

**Figure 6.**
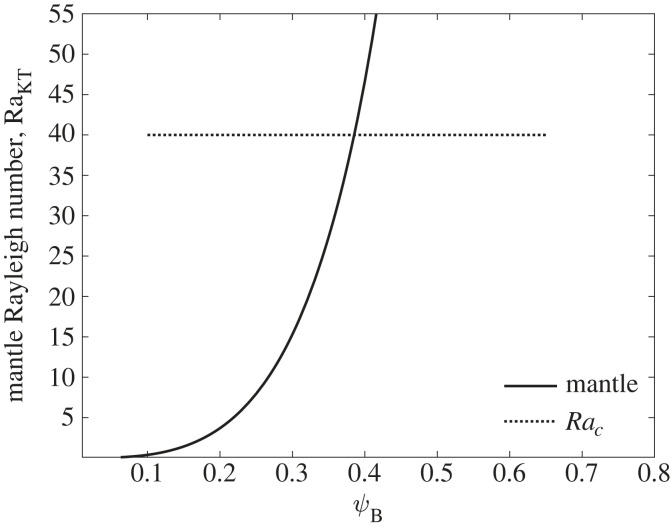
Raleigh number versus porosity for temperature differences mantle inner to mantle outer, with critical Raleigh number line *Ra_c_* = 40.

Using the parameters in table 2, mantle Rayleigh numbers were calculated for the temperatures found in the cluster mantle inner to mantle outer, T_0_ = 283 K (10°C), T_1_ = 291 K (18°C) as shown in figure 6.

**Table 2.** Convection conduction transition parameters.

symbol	value	source
$m_{B}$	$115\times 10^{-6}\;\mathrm{kg}$	[27]⁠
$l_{B}$	$13\times 10^{-3}\;m$	[27]⁠
$d_{B}$	$4.5\times 10^{-3}\;m$	[27]⁠
$Cp_{B}$	$3.3\times 10^{3}\;{\mathrm{Jkg}}^{-1}\;K^{-1}$	[48]⁠
$k_{B}$	$0.5\;{\mathrm{WK}}^{-1}\;m^{-1}$	§4.2
$\rho_{\mathrm{Ah}}$	$200\times 10^{6}\;m^{-2}$	[49]⁠
$\;p_{h}$	5	[49]⁠
$d_{h}$	$3\times 10^{-6}\;m$	[49]⁠
$l_{h}$	$0.75\times 10^{-3}\;m$	[49]⁠
$\bar{L}$	$10\times 10^{-3}\;m$	[45]⁠

The results indicate that at porosities in the mantle and core of the cluster, natural convection either does not occur or only at very low velocities. Therefore for heat transfer purposes the mantle and core can be treated as solids with the effective conductivity related by equation (2.7). Further, pre-cluster state convection is likely to be weak as this has a porosity close to 0.5, especially if the hive is of low thermal conductance, with low internal temperature differences.

### 4.2. Honeybee body, pre-cluster, core and mantle thermal conductivity

**Figure 7.**
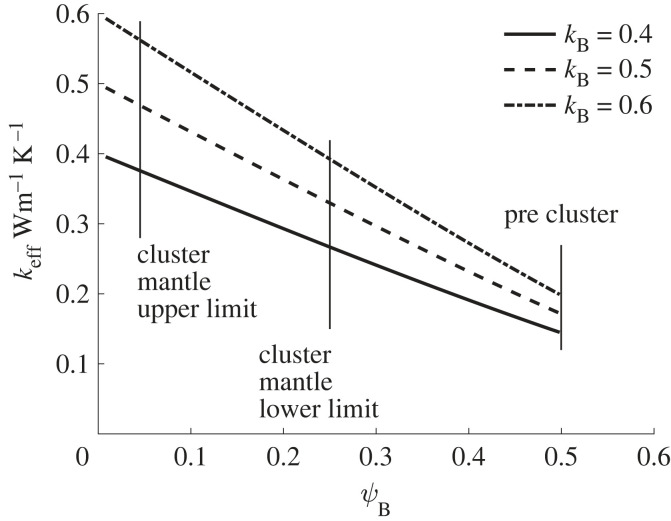
Effective conductivity *k*_eff_ of bee/air mixtures versus porosity *ψ*_B_ using EMT, where *k*_B_ = 0.4, 0.5, 0.6 and *k*_air_ = 0.026.

Because of the experimental uncertainty and the methods used by experimenters in table 3, the range of honeybee conductivities for 0.4 to 0.6 W m^−1^ K^−1^ will be considered. The variation of effective conductivity with porosity is shown in the graph in figure 7. This results in an effective thermal conductivity of the mantle when not clustered (porosity 0.5) [27]⁠ of approximately 0.2 W m^−1^ K^−1^ and when tightly clustered (porosity 0.05 [44],⁠ i.e. sphero-cylinder packing limit with hair) of approximately 0.5 W m^−1^ K^−1^. This also shows that there is an increase in conductivity by approximately 2 in the transition from not clustered to tightly clustered over a wide range of honeybee body conductivity (from 0.4 to 0.6 W m^−1^ K^−1^) and mantle porosity (0.25 to 0.05). Therefore the cluster mantle does not meet the second criteria (*δk*_mantle_/*δΓ*) < 0 ⇒ insulator, i.e. conductivity definition of ‘insulating’.

At lower ambient temperatures, the cluster core has been shown and described by many researchers to be of a lower density or higher porosity than the mantle, but of less porosity than the pre-cluster state [41]⁠. Thus we can infer that, for heat transfer, the core can be treated as a solid, and that from figure 7, we can reliably infer that the thermal conductivity of the mantle is higher than that of the core. Therefore the third criteria *k*_mantle_ < *k*_core_ ⇒ insulator for insulation is not met and instead fulfils the criteria for a heat sink.

### 4.3. Heat loss, mantle size ratio determination

**Figure 8.**
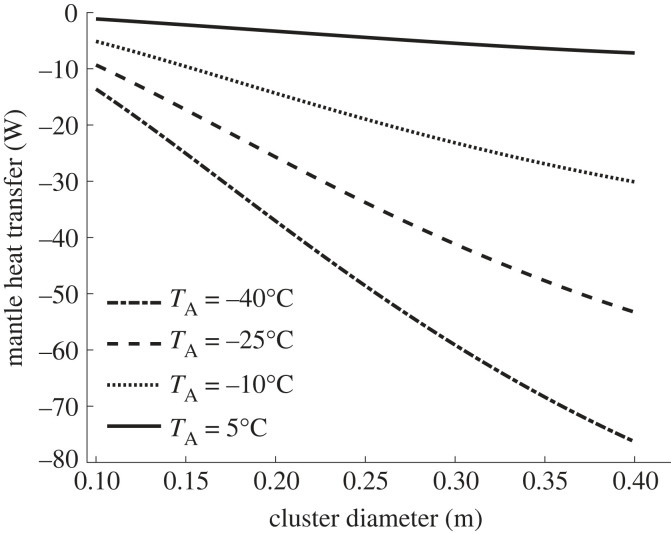
Cluster radius versus mantle heat flux (negative is heat loss) for varying air temperatures *T*_a_ in a bare landscape inside British National wooden hive with 19 mm walls conductivity 0.12 W m^−1^ K^−1^. Effective sky temperatures derived for 1% humidity.

Using the principle of the conservation of energy this can be represented by a set of three nonlinear equations using convection correlations [36,50]⁠, and radiative heat transfer rules [51]⁠. These can be iteratively solved [42]⁠ to yield the mantle heat flux, i.e. heat loss, and hive surface temperatures from a known size of cluster, hive properties and outside conditions, as shown in figure 8 (see Methods).

For all outside conditions heat transfer varies with mantle size at a rate between −70 and −450 W m^−1^, i.e. $(\delta \dot{q}/\delta r)<0$. This means that the mantle does not fulfil the first criteria, i.e. heat loss versus size criteria for insulation, and instead acts like a heat sink.

### 4.4. *R*-value analysis

**Figure 9.**
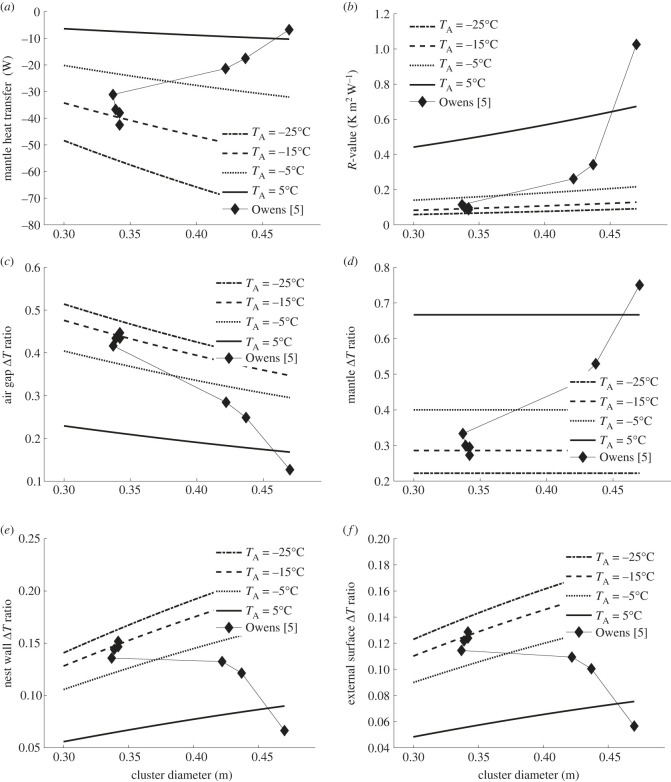
For isotherms from +5°C to −20°C and the values of temperature and cluster size from [5] versus, (*a*) metabolic heat, (*b*) *R*-value, (*c*) internal hive air gap Δ*T* as proportion of mantle to ambient Δ*T*, (*d*) mantle Δ*T* as proportion of mantle to ambient Δ*T*, (*e*) hive wall Δ*T* as proportion of mantle to ambient Δ*T* and (*f*) external surface to ambient Δ*T* as proportion of mantle to ambient Δ*T*. Using the same size hive as [5] shaded from the sky in still air.

In figure 9, for isotherms from +5°C to −20°C and the values of temperature and cluster size from [5], the results from the model in §3.3 were plotted against (*a*) metabolic heat, (*b*) *R*-value, (*c*) air gap temperature difference as proportion of mantle to ambient temperature difference, (*d*) mantle temperature difference as proportion of mantle to ambient temperature difference.

**Figure 10.**
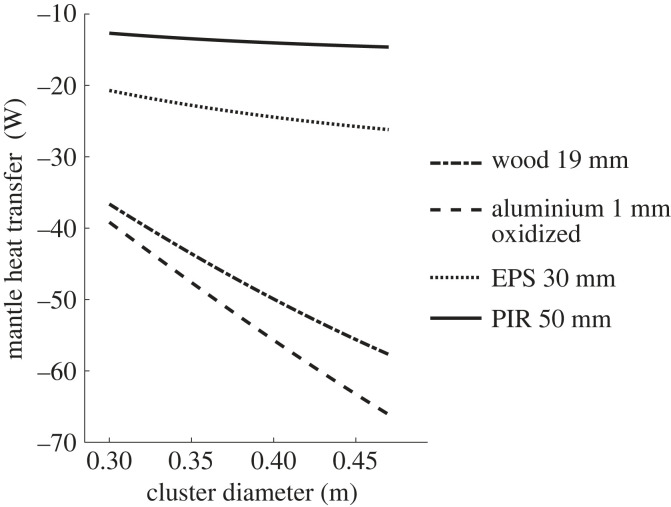
Metabolic heat versus cluster diameter *T*_A_ = −16.7°C for various wall materials. Using the same size hive as [5] shaded from the sky in still air.

For material comparison, the mantle heat transfer was plotted (figure 10) for various hive materials at 16.7°C (wood 19 mm *k*_hive_ = 0.1, *ε*_outer_ = 0.9; aluminium 1 mm oxidized *k*_hive_ = 200, *ε*_outer_ = 0.2; polyisocyanurate (PIR) 50 mm, *k*_hive_ = 0.023, *ε*_outer_ = 0.3; expanded polystyrene (EPS) 30 mm), *k*_hive_ = 0.03, ε_outer_ = 0.9).

We can see that the cluster diminishes in diameter from 0.47 to 0.34 m when the temperature falls from 6.7°C to −16.7°C with the reduction in diameter ceasing at approximately −10°C at approximately 0.17 m. This is reflected by an increase in metabolic power from 6.8 to 42.5 W and a reduction in *R*-value from 1.33 to 0.11. Over the same interval the proportion of the core to temperature difference supplied by the mantle falls from 80% to 33% while the air gap between the mantle and the hive inner surface grows from 10% to 41%.

The heat loss from the colony of approximately 36 W when the temperature drops from +6.7°C to −16.7°C gives a gradient of approximately 1.53 W K^−1^, which compares with a previously found gradient of 0.57 W K^−1^ kg^−1^, which would suggest Owens used a colony of approximately 2.7 kg, a realistic value for wintered colonies in that locale [40,52]. Unfortunately we do not know actual weight of the colony.

This means that the mantle does not fulfil the fourth criteria to be an insulator.

## 5. Discussion

The cluster mantle does not meet any the four insulation criteria identified and meets all three heat sink criteria.

So why cluster? Honeybees need 25°C to be at their best for heat producing, below 18°C their heating ability falls fast, and at 10°C they are on the edge of life [53]⁠. So if the inside surface of the hive is 18°C+ (i.e. the 18°C contour is close to or in the hive walls), and the bees are comfortable producing the heat required to maintain it (figure 1*a*), then it is thermally like a summer swarm cluster [25]⁠, which rests with its periphery regulated to 18°C. This then requires little additional heat or bees to raise local temperatures to brood heat 34°C [54]⁠, as the brood is sitting in a volume in which convection is suppressed, with lower conductivity, and among honeybees able to deliver extra metabolic heat.

Once the outside temperature falls, the heat needed to sustain 18°C+ inside goes up. If it goes above sustainable heat production level, things start to happen. The temperature near the hive wall drops and so does that of the honeybees near it, when they rest from heating. For the individual bees which have been chilled, they have to get closer to honeybees that can still effectively produce heat, so they move inwards from the hive walls [4,25]⁠. This creates a bee-less gap next to the wall. As the outside temperature falls further, this eventually makes this 18°C contour move towards the centre of the hive (figure 1*b*). Convection currents occur in the growing gap between the hive walls and the honeybees. This increases the heat loss. The honeybees get closer together and their conductivity increases, while improving the survival of the outer honeybees, makes the heat loss greater. Now some of the core honeybees are cooled below 18°C, so more of those shut down, and the collapse inwards continues. The outside conditions worsen and the 10°C contour now enters the hive internal space along with stronger convection currents (figure 1*c*). Honeybees that stay outside this contour will die.

Where this 10°C contour lies now determines the thermal environment. The heat is now being produced by a few bees inside the 18°C contour that are at high levels of exertion. These produce heat for a short time and are then replaced by other honeybees [4]⁠. This shrinking of the cluster and reduction of the core goes on until heat passing through the surface area of bees at 10°C is reduced, to be in balance with the ability to produce heat by honeybees remaining at 18°C and above. As a consequence, the total heat production of the colony and the level of all other activity has collapsed [40]⁠. Now instead of unstressed bees, we have bees alternately stressed by low temperature and high exertion.

In order to maintain mantle surface temperature, once the maximum density of the mantle is reached, further decreases in ambient temperature require one or more of the following: the mantle thinning by expansion of the core; the mantle thinning by honeybees on the outside dying and falling off; the core increasing its temperature.

In anthropomorphic terms, clustering as described above is not a ‘wrapping of a thick blanket’ to keep warm, but more like a desperate struggle to crowd closer to the ‘fire’ or otherwise die and fail the colony. Calling it an insulator gives a false impression of its role in the nest. A more accurate descriptive term may be ‘increased conduction mitigated by domain collapse’.

This is not the currently accepted view. This was shaped, first by Phillips & Demuth [13] and then Farrar [14,24], who commented that the cluster provided its own insulation, and then later by the work of Southwick who describes, in several papers, clustering as an increase in thermal effectiveness [16,41]⁠⁠, and stresses the close packing of the honeybees increasing the thermal resistance through interlocking hair and therefore several packed layers of bees as an effective insulating coat for the cluster. This led to some assuming that the thermal conductivity decreased with increasing density [28]. Unfortunately this is incorrect as convection is suppressed when they are dispersed at a porosity of a round 50% i.e. approximately 1 to 2 mm between bees [27]⁠, close to their pre-cluster state. At the densities Southwick refers to, the cluster is mostly honeybee and not air.

The difference in view resides primarily in the knowledge or assumption of pre-cluster state. Convention has used the long-held assumption that the pre-cluster state is one where high-value convection is dominant, and clustering reduces the convection around the individual honeybees and replaces it with low-value conduction.

However recent research [27]⁠, shows that the pre-cluster state is one of low-value conduction and weak convection, which, on clustering, is replaced by high-value convection around the cluster and doubling of the conduction within, which necessitates a dramatic partial shutdown of core heat production and other activities, offset by a reduction in surface area and an increase of stress on the individual honeybees.

In addition, some attribute the bulk of the core to ambient temperature difference to the properties of the mantle [20] giving as a reason the small temperature difference between the hive inner and outer surfaces. This is evident not only in direct statements [20], but also in the comparing of body weight to heat conductance of bee colonies with that of other animals. Those animals' weights include the weight of the structure causing the temperature difference (i.e. fur, feathers), but the structure for the honeybees (i.e. the hive) has been omitted [41]. However, they have overlooked the high proportion of the heat difference being provided by the hive cavity (air gap, figure 9*c*). As the temperature drops the share of the temperature difference moves from the mantle (approx. 33%) to the air cavity (approx. 40%) and to a lesser extent the hive wall (approx. 14%) and external surface air (approx. 12%). This air gap makes this wooden hive's performance not substantially different to one of metal (figure 10) [20,55].

This can be summarized as either overlooking, or misunderstanding the complex interaction of the colony enclosure and thermofluids (heat, radiation, water vapour, air) with honeybee behaviour and physiology, i.e. not recognizing the enclosure as within the extended phenotype.

As regard the limitations of the model presented:

— The relation used here for heat transfer of the mantle to the cavity, is that of a sphere and not an ellipsoid interrupted by thermally conductive combs. However this is only likely to change the magnitude and not direction of the *R*-value, as the observed increase in heat loss and decrease in surface area of the cluster will prevail.
— Owens had ventilation holes piercing the hive shell, if this had increased the rate of heat loss, one might have expected an increase in colony size (see §4.4). Yet the predicted colony size is realistic for the locale [52]. This is explained by such holes having little thermal impact in high thermal conductance enclosures [7].
— In determining the onset and magnitude of convection in the porous cluster, current heat transfer relations used here do not explicitly reflect: the isothermy of the core, the isothermy of the mantle outer surface and the boundary effects of the narrow space between the combs. Further, the size of the honeybee being approximately half the gap between the combs adds further complexity. Thus the relations used are a simplification of a complex expanding subject [39,56].
— Anisotropy resulting from hair distribution.

All of which are subjects for further research.

## 6. Conclusion

All substances can create a temperature difference. The use of the word ‘insulation’, in connection with clusters, means more than that. It implies, in this case an unwarranted, positive value judgement about the substance or configuration and has, with its repetition, influenced interactions with honeybees, encouraging practices of using thin-walled wooden hives and the North American refrigeration of honeybee colonies.

This study has shown that, in any reasonable interpretation of the word ‘insulation’, the clustering process results in its decrease and that a cluster is an increase in conduction, mitigated by collapsing the colony domain. A transition from a state where the honeybees can suppress internal convection within the nest, into a state of high internal convection and conduction, results in increased individual honeybee stress. This is opposed to the conventional view that the cluster is a benign thermal improvement on the pre-cluster state.

The conventional view does not match the recent advances in research, and enables an avoidable increase in honeybee stress, (i.e. refrigeration and use of hives not significantly different in performance from thin metal), when they are facing unavoidable increases in stress from pests, disease and climate change.

Imposing avoidable stresses on vertebrates by provoking behavioural survival responses for no benefit to the individual or groups of animals may be regarded as cruelty. Although present ethics standards for insects are different, changes in practice that reduce the frequency and duration of clustering should be urgently considered, researched and promoted (e.g. using hives from materials in figure 10).

## Data Availability

Supplementary material is available online [57].
